# Infrastructure development and environmental risk perceptions in the Wild Coast, South Africa

**DOI:** 10.4102/jamba.v15i1.1377

**Published:** 2023-07-18

**Authors:** Tafadzwa Mambiravana, Ikechukwu Umejesi

**Affiliations:** 1 Department of Sociology, Faculty of Social Sciences and Humanities, University of Fort Hare, East London, South Africa

**Keywords:** risk perceptions, Wild Coast, road project, local communities, infrastructure

## Abstract

Government’s proposal to construct the N2 Toll Road in the Wild Coast was lauded for its ‘developmental agenda’ in the historically neglected Wild Coast communities of the Eastern Cape province. This project, the government and business groups envisaged, would open up the coastal communities of the Eastern Cape and KwaZulu-Natal provinces to economic development of the region and the nation in general. However, the road project has equally been criticised by several local communities and environmental advocacy groups who associate the road with the controversial plan to mine titanium in the region and its anticipated social and ecological disasters. Using a qualitative research approach that utilised face-to-face interviews, focus group discussions, secondary data review and observations, the study found that different communities associate the project with high risks regarding their environment.

**Contribution:**

The study was anchored on the cultural theory of risk perception, which helped in exploring how people’s preferences differ in terms of how life should be organised, their perceptions of risk, and their responses to it.

## Introduction

While successive post-apartheid governments in South Africa view the N2 Toll Road project in the Wild Coast as critical infrastructure that will benefit local communities and the nation in general, local community members and various environmental advocacy groups view it as something that will have a negative impact on the environment. Stretching between East London in the Eastern Cape province and Durban in the KwaZulu-Natal province, the 550 km road project, together with the Pondoland National Park, have been identified as the government’s Wild Coast Spatial Development Initiatives^1^ (SDI) aimed at combating poverty, generating employment, and uplifting the impoverished communities of the Wild Coast region (Draft Environmental Impact Report Proposed N2 Wild Coast Toll Highway 2008). The government envisages that the road will become a gateway to the richly endowed Wild Coast region and will foster greater accessibility and economic development through mining, tourism, forestry, agriculture, and other developmental activities. The centrality of road infrastructure to the achievement of these goals is demonstrated by the South Africa National Road Agency Limited (SANRAL) spokesperson when he was speaking on behalf of the state during the Human Science Research Council (HSRC) Survey in 2019. He said:

‘The wild coast Toll Road Project will make a major contribution to redress wrongs of the past and become a catalyst for development for the purpose of Pondoland. It offers cheaper and safer transport and enhanced access to healthcare, education, services and jobs. For the Eastern Cape, it will unlock opportunities in the field of eco-tourism, agri-processing and trading through improved access to the broader transport network.’ (Daily Maverick 19 March 2019:1–3)

According to the Department of Transport (2018), such a targeted development is aimed at South Africa’s economic transformation agenda, modernisation of the economy, as well as bringing it in line with the Freedom Charter, the national Constitution and the National Development Plan (NDP). However, the N2 Toll Road project has attracted diverse reactions since its inception in the early 1990s to date because of different views among various stakeholders. Over time, risk perceptions have also been brewing because of a lack of robust consultations with local communities and inadequate social and environmental impact assessment. Although construction is underway, numerous communities along the Wild Coast have staged a series of protests, halting progress on the project and delaying its completion. These communities together with environmental advocacy groups in the region are concerned that the road project would lead to environmental degradation and destruction of rare plant species and sacred aquatic environments, among other forms of environment. More so, these groups fear that the road construction would lead to loss of land, change their way of life and facilitate the mining of titanium and dunes (see Healy 2022). Although there is vocal opposition against the road project, the state and business groups are profoundly supportive of the project. Most importantly, it is in this conundrum that this study seeks to contribute to knowledge, the understanding of risk analysis by governments in grassroots development, and inclusivity as the basis of sustainable rural development.

## Infrastructure development and risk perceptions in the Wild Coast: The contention

The emergence of democracy in 1994 and the subsequent entrenchment of democratic ideals in the post-apartheid South Africa necessitated massive infrastructural developments, particularly in the former homelands where developmental projects and programmes were previously ‘deliberately’ prevented, except for those projects that served the economic interests of the colonial or apartheid governments (Gumede 2017; Oldfield & Greyling 2015). Thus, the need for the construction of the N2 Toll Road was informed by the history of institutionalised marginalisation of the former black (Bantustans) homelands during the apartheid^2^ era (De Wet 2013).

However, there have been diverse views over the N2 Toll Road project since its inception in the late 1990s because of perceived environmental risk perceptions of different stakeholders. For instance, while the state and the construction firms hold the view that the road would transform the local economic situation of the region, various local communities who oppose the construction of the road see it as an invitation to socio-ecological disaster, a view equally shared by the environmental advocacy groups (Ntshona & Lahiff 2003). Therefore, this study sought to understand how the different stakeholders linked to the N2 Toll Road project perceive risks associated with the project. It is imperative to unpack these risk perceptions as a way of understanding the festering conflicts between various stakeholders, particularly the state and local communities along the N2 Toll route.

## Infrastructure development and environmental risk perceptions: A critical review

Infrastructure is fundamental for development because it improves people’s standards of living by providing access to the labour market, education, healthcare and other important social services (Thacker et al. 2019). Infrastructure also plays a critical role in establishing rural and urban links through connecting rural territories to regional and international networks as well as bringing about inclusive and long-term changes in the production, institutional and social spheres (Simone 2014). Given this, infrastructure has been incorporated into the Sustainable Development Goals of the 2030 Agenda of the United Nations as a crucial tool to improve living conditions, promote greater social stability and create cities and territories that are more resilient to climate change (United Nations 2015).

However, massive infrastructure projects such as road and dam construction are increasingly causing direct and indirect impacts on the environment (Laurance et al. 2015). More so, infrastructure types also differ in terms of how they produce cumulative effects by facilitating successive developments and changing the behaviour of other entities interacting with the landscape (Laurance et al. 2015).

Apart from direct impacts, infrastructure development causes indirect effects such as poaching, logging, colonisation and other human incursions; it is thus critical to ‘avoid the first cut’, particularly in pristine regions (Teo et al. 2019). In light of the direct and indirect environmental impacts stated here, factors that influence lay people’s risk perceptions and judgments need to be explored in order to establish successful communication between the public and the government.

Before exploring this subject further, it is imperative to give an insight into what is risk and what risk perception entails. Risk as defined by Giddens (2002:22) refers to a danger that is ‘actively assessed in relation to future possibilities’. Scholars such as Beck (2005), Giddens (2002) and Castels (2006) have widely written about risk society, a society that is epitomised by the social hierarchy that is increasingly based on risk, rather than on wealth. They argue that risk society focuses on the distribution of ‘bads’ rather than the distribution of ‘goods’. Castels (2006) also links the aspect of risk and the concern of governing people who have been marginalised or excluded because of gender, class, race and other bases of social inequality, of which the marginalised communities of the Wild Coast in this study, is a case in point.

Sjoberg (1996) suggests that risk perception can be contrasted with ‘real risk’ or the actual damage from a hazard, in that it refers to an individual’s or population’s judgement of the hazard and its risk. In contrast to ‘real risk’, there are three issues to take cognisance of. Firstly, while different from real risk, the aspect of probability exists in perceived risk mainly through biases (Siegrist & Gutscher 2006). Secondly, in perceived risk, the level of uncertainty or potential severity of the event outcomes reflects danger to different groups and individuals depending on their preferences and coping capacities. Lastly, there is the social construction of risk, which refers to the level of danger that society is ready to take in exchange for the social advantages associated with the cause of a relationship that is influenced by views of who bears the risk mitigation burden (Sullivan-Wiley & Gianotti 2017).

Although higher levels of risk perception are most likely to lead to protective action, Wachinger et al. (2013) argue that this relationship is not straightforward because of a phenomenon called the ‘risk perception paradox’, which proposes that high risk perception is not always linked to protective action. The authors posit that the difficulties in translating risk perception to action may be pertinent in multi-hazard contexts where people are subject to several overlapping dangers and have limited resources to deal with them. Many scholars, for example, Goyal and Gurtoo (2011) and Siegrist and Hartmann (2020), have investigated how people perceive risks associated with technological hazards such as genetically modified organisms and nuclear power. Our study shows that risk perception is subjective, as it depends on the individual’s or group’s attributes as well as on the nature of the danger itself.

Environmental risk perceptions linked to infrastructure development are evident in the construction of dams in Malaysia (Aiken & Leigh 2015). In Malaysia, there has been a proliferation of dam construction in the past two decades to supply water to urban areas, deliver energy and facilitate irrigation. These developments came at a high price in terms of environmental impacts such as loss of habitat and biodiversity, depletion of fish stocks, obliteration of food security and forced displacements. For Malaysia’s populations such as the Orang Asli and the inhabitants of Sarawak and Sabah, there were fears that livelihoods would be severely affected as they largely depended on hunting, shifting cultivation, fishing and gathering for subsistence and trade (Aiken & Leigh 2015). As a result, the environmental costs for these communities are significantly higher, considering that the majority of the project’s benefits, such as energy and water supplies, normally accrue to distant urban populations.

At a regional level, the Maputo Corridor^3^ is one of the mega infrastructure development projects in Southern Africa associated with various risk perceptions. Despite its goal to improve transport and lower barriers to trade, the project is substantially linked to risk perceptions such as fear of exclusion among ordinary citizens, fear of self-interest among government officials and distrust or concerns about ceding power or developing a level of dependence to a broader external constituency (see Hagerman 2012). These risk perceptions have resulted in inevitable resistance to change, despite the fact that initiatives such as a One-Stop Border Post^4^ (OSBP) can benefit both truckers travelling across borders and the countless formal and informal industries that have established themselves based on the level of efficiency that the OSBP seeks to improve (Hagerman 2012).

Although there are massive infrastructure projects taking place throughout Africa including the Maputo Corridor mentioned here, there is paucity of literature on infrastructure development and environmental risk perceptions. Therefore, our study sought to cover this research gap by exploring environmental risk perceptions linked to the N2 Toll Road project in South Africa’s Wild Coast region.

## Theoretical framework

**FIGURE 1:**
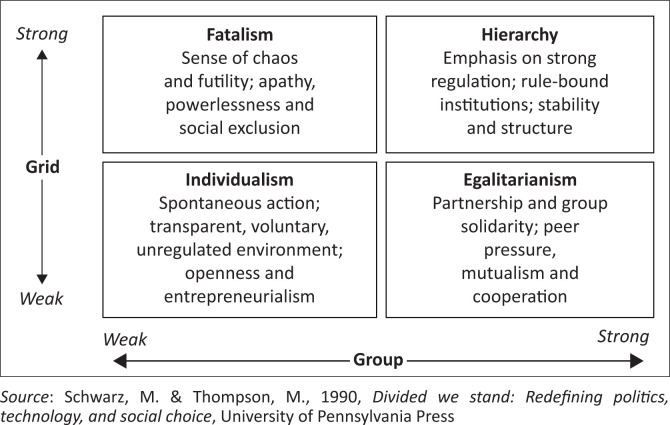
Grid/Group dimensions of sociality, based on Thompson et al. (1990).

The cultural theory (CT) of risk perception seeks to explain how people perceive and understand risk (Douglas & Wildavsky 1983). Various studies (Brenot, Bonnefous & Marris 1998; Yuan, Zeng & Swedlow 2020) corroborate the CT’s assertion that there is a strong link between cultural world views and risk perception. Lieske, Wade and Roness (2014) and Lin et al. (2018) observe that people’s perceptions of environmental risk increase their sense of urgency and obligation to safeguard the environment, which leads to increased environmental protection practices. The use of CT’s ‘grid-group’ framework helps us understand how four different social groups with different core values influence people’ perception and values (Douglas 2007). The ‘grid-group’ entails four types of risk culture (Figure 1), namely hierarchy, egalitarianism, individualism and fatalism.

As indicated here, the four groups, namely the hierachists, the individualists, the egalitarians and the fatalists have unique and specific worldviews, which determine their understanding and interpretation of risks and vulnerabilities in social ecological discourse. Individuals and groups aligned to an egalitarian orientation hold the belief that everyone in society is equal (Chen, Cheng & Urpelainen 2016; Swedlow 2014), and they are more likely to accept ideas that advocate for equal and fair treatment of humans and nature (Jenaro et al. 2005). For instance, egalitarians focus their attention on environmental and social advocacy for the development of both human society and the natural environment in the common good. Therefore, they are equally concerned about environmental risks and environmental protection (Zeng, Liu & Yi 2019).

In contrast, individuals and groups that hold an individualistic orientation are more concerned with freedom than with equality. They are not much concerned about environmental risks (Hermand et al. 2003). Individualists perceive environmental regulation and protection as threats to development of industry and businesses, and this viewpoint will further reduce their pro-environmental behaviour (Hermand et al. 2003). Therefore, we argue that construction firms and business people in this study hold the individualist orientation.

Individuals and groups who hold a hierarchical orientation or culture believe that everyone in society has their own role and status (Lachapelle & McCool 2007) and environmental risk is seen as a ‘hidden symbolic cultural model of social elite status and authority’. They have trust in authority and they hold the view that excessive concern about environmental threats is a threat to the status quo. Thus, they are more likely to place a low value on environmental protection, to not perceive environmental risk, and to not engage in pro-environmental behaviour. However, some environmental risk perception discourses such as climate change (McEvoy et al. 2014) have indicated that hierarchists and egalitarians are closely aligned. Thus, in case of the N2 Toll Road project, the state relies on environmental experts in order to implement sound environmental policies.

Lastly, fatalists, in this case grassroot communities, have been shown to have little to do with environmental issues and in most cases do not maintain a stable position. They feel excluded in environmental policy decisions and they believe that the environment is inherently at risk (Chen et al. 2016). The fatalists hold the view that there is no fairness in environmental discourse as they perceive that they are excluded in decision-making. Marginalised ordinary people in communities are more likely to hold this view. In short, the assessment of the theoretical framework has led to a discussion of some environmental issues, namely loss of habitat and rare plant species, destruction of sacred aquatic environments and cultural spaces, removal of graves, land degradation and environmental pollution. What prompted this study was that despite various Environmental Impact Assessments (EIAs) conducted for the project (see EIA reports 2003, 2007, 2016), fears of risks and vulnerability linked to the N2 Toll Road project have delayed its launch and caused construction activities to be halted in some areas. The praxis of the aforementioned worldviews on the N2 Toll Road is detailed in the discussion section.

## Materials and methods

This article used a qualitative research design comprising 20 semi-structured interviews administered to local communities and individuals affiliated to environmental advocacy groups in the region where the N2 Toll Road is scheduled to take place. Data were collected between July and August 2021. The participants were given pseudonyms for ethical reasons. Furthermore, the study was conducted during the COVID-19 pandemic, and the researchers complied with COVID-19 regulations proclaimed by the Department of Health.

## Setting

The Wild Coast region is located in the Eastern Cape province of South Africa. The area lies between the Great Kei River in the South and the Mthamvuna River in the North. The Drakensberg and Stormberg mountain ranges are to the west. Geographically, the study area covers 42 240 square kilometres (km²). The estimated population is 1.4 million people and population density is estimated at 96 people per km² (Goliath, Timla & Mxunyelwa 2018). A huge part of the land in the Wild Coast is called Pondoand,^5^ which is well-known because of the ‘Pondoland revolts’ of 1958–1960, when there were widespread protests against the introduction of Bantu (Tribal) authorities, Bantu education, and the so-called ‘betterment’ policy of forced removal and zonation (Kepe 2011).

This study was centred on a small area called Msikaba in Lusikisiki where the N2 Toll Road is expected to pass through. A huge bridge is being constructed in this area. The people from this area hold diverse views regarding these ongoing infrastructure developments, with the majority arguing that it is a precursor to titanium mining activities of the dunes in Xolobeni.

### Sampling technique

A purposive sampling technique was used to select 20 participants from Msikaba area in Lusikisiki. The participants included 12 environmental experts and 8 local community members. This sample represents the broader populations in the region who are affected by the project, such as farmers and local landowners although many of them are environmental experts. Semi-structured interviews were conducted with the participants and were triangulated with secondary data obtained from a desktop review. Documents related to land and environmental policies were used to establish the environmental risk perceptions associated with the N2 Toll Road project and to determine the gaps and weaknesses for further consideration.

### Data analysis

Data gathered from in-depth interviews were transcribed and are presented according to the objectives of the study. The study also used secondary data such as environmental reports, which were analysed using content analysis. According to Neuman (2006), content analysis is a method for gathering and analysing textual content such as meanings, words, symbols, pictures, ideas, themes, or any other message that can be communicated.

## Results and discussion

### Loss of habitat and rare plant species

During interviews in the Wild Coast in the Bizana area, the researchers spoke to one of the environmental experts affiliated to Wildlife and Environment Society of South Africa (WESSA). The participant exhibited rich knowledge about how the road construction would affect the environment, especially in Pondoland. The participant (E1) said:

‘The road will pass through the Pondoland centre of endemism, which forms the part biodiversity hotspot, one of the 36 hotspots that are found globally. It lies within the Maputaland-Pondoland-Albany Hotspot. The area is very small and it covers an area of about 1885 km^2^. It is an area that constitute about 1 per cent of the global diversity hotspots and has got about 200 plants that are endemic to Pondoland and not found anywhere in the world. The unique thing is that the plants are very rare for example Pondoland Conebush and Pondo Coconut, which is found between Mnyameni River and Kutume River near Mbotyi. Therefore, if constructing a highway in such a sensitive area wipes out all the magnificent species. Therefore, this road actually threatens the Pondoland centre of endemism.’ (Interview with Participant E1, 29 July, 2019)

The given narrative also concurs with the Draft Environmental Report (2008), which anticipates that the wide road will result in the direct loss of some habitat adjacent to the already existing road, including habitats associated with the Mthatha Moist Grassland, Bisho Thornveld and Eastern Valley vegetation types. In light of the environmental concerns highlighted here, a number of appeals were made objecting to the authorisation of the road project. Evidently, environmental advocacy groups such as the WESSA and Botanical Society of South Africa (BSSA) argue that the project should comply with the relevant requirements of the *Environmental Conservation Act* (*ECA*) *of 1998*, and the *National Environmental Management Act of 1998* (*NEMA, Act No. 107 of 1998*) (Huggins, Andrews & Zigel 2008).

### Destruction of sacred aquatic environments, cultural spaces and removal of graves

The N2 Toll Road project faced opposition from some institutional stakeholders such as environmental advocacy groups and local communities because it is most likely to lead to the destruction of sacred places, aquatic environments and the removal of graves. The protection of these elements by the local communities has created tensions between local communities and the state. The participants stressed that these elements reflect a close bond between villagers and their ancestors. They also pointed out that the destruction of these sites is a threat to local values and culture and no form of compensation would restore this bond.

In Msikaba, the researchers interviewed a participant, approximately 70 years old, whose father was a Sangoma.^6^ The researchers asked the participant about the encroachment of the road on places such as rivers and streams and what this meant for those spaces. The participant, here referred to as Mr Xuma, said:

‘All the places that you have mentioned play a significant role in our community’s culture and beliefs. We actually regard the water from our rivers as the most pure water and can be used to bathe or to sprinkle around the houses as a way to chase off bad spirits or demons. Here in the Wild Coast, we have two places that are popularly known and these are Isinuka in Port St Johns and Sibhenga near Lusikisiki where people visit and bath themselves and carry the water for home cleansing. Therefore, in my own view, road construction will greatly affect our important spaces in many ways. New people from other places will not respect and value these spaces the way we do.’ (Interview with Participant C1, 30 July, 2019)

To be acquainted with the practices in the sacred sites mentioned here, the researchers requested one of the tour guides in Msikaba to accompany them to Isinuka in Port St. Johns, 100 km away from Msikaba. During an interview with this tour guide, here referred to as Joe, it was stated that:

‘Construction companies are most likely to dump waste in the rivers and pollute the water, which we value so much in our culture. If the river is polluted, this affects the people who use it for cultural rituals and their other needs.’ (Interview with Participant E2, 03 August, 2021)

Similarly, another tour guide in Port St. Johns who was also an environmental expert explained that his aunt had been trained in the river to become a traditional healer. The tour guide, here referred to as Sue, explained that:

‘Sometimes a person can get a vision of the river, meaning that they need to be trained in the river for them to become a traditional healer. When you are a traditional healer trained in the river, you can go into the river with them and can even spend up to thirty minutes in the river, underwater.’ (Interview with Participant E1, 04 August 2021)

In Isinuka, Port St. Johns, a local community member, Mrs Toko who was adamantly opposed to the N2 Toll Road project, agreed with the views captured here. She noticed that rivers and streams are necessary for ritual cleansing and healing. The participant stated:

‘We believe that if you bath in that water you will come out clean from bad luck and evil spirits. The water also heals those that are sick. Once you fall sick, you can come and take a bath in this river and all your ailments will disappear from you.’ (Interview with Participant C3, 07 August 2021)

The given narratives show that communities in the Wild Coast are connected with their aquatic environments through a variety of cultural and belief practices. These practices have a spiritual and aesthetic significance in their lives. Thus, the participants were concerned that the construction of the N2 Toll Road may jeopardise their cultural and religious practices. This is similar to the findings of Mboweni and De Crom (2016) in a study on the ‘narrative interpretations of the cultural impressions on water of the communities along the Vaal River, Parys, Free State’. Their study found that the location of the Vaal River and its association with holy beings in certain cultures and beliefs are primarily what results in the religious practices that are carried out at rivers.

The researchers also asked the participants about the potential risks of the N2 Toll Road project on graves, initiation sites and other sociocultural heritage sites. A curator elaborated that the risks are significant but mitigation measures are being put in place to secure local culture and avoid the disruption of sociocultural heritage. She said:

‘The road of course has impacts on family and ancestral graves, initiation sites and as well as socio-cultural heritage. Local people are very much afraid that the road construction process, especially the influx of people from different places, will result in the distortion of their traditional culture and disrupt their way of living characterised by communal values and beliefs. However, there is a task team that was selected to research about all the potential impacts of the road including the environmental impacts.’ (Interview with Participants C5, 08 August 2021)

The given statement of the curator concurs with the Cultural Heritage Impact Assessment report published in 2016. The report states that the project would result in many potential impacts related to historical, cultural heritage and archaeological sites. The participants stressed that the graves also reflect a close bond between villagers and their ancestors. During the interviews, a villager said:

‘We respect our ancestors a lot and we believe they play a critical role in our daily lives, hence we will not allow anyone to come and tell us what to do with our ancestral graves. No compensation or amount of money will be enough to pay for the removal of our ancestral graves. We communicate with them and believe in their blessings as Africans. Therefore, destroying or relocating their graves is a total disrespect for them and we do not know the consequences of that.’ (Interview with Participant C6, 09 August 2021)

The views of the given participants show that the N2 Toll Road construction would result in displacement of people, removal of graves and destruction of cultural heritage sites in some places. The majority of the participants interviewed were vehemently against the removal of graves, claiming that these constitute a strong cultural significance in their lives. The views of the participants are consistent with the findings of Saccaggi (2013) in a study entitled ‘Disenfranchised heritage: Ancestral graves and their legal protection in Limpopo, South Africa’. In Saccaggi’s study, a group of residents of Blinkwater farm in the Mapela area was relocated to Sekuruwe Village by the Potgietesrus Platinum Limited, a platinum mine operating company in the province of Limpopo. Before they were relocated in 2006, these residents had been staying at Blinkwater farm for more than 100 years. During the relocation, approximately 150 of their ancestral graves were exhumed from the traditional land and moved to the new cemetery near Sekuruwe village. According to Saccaggi (2013), Sekuruwe’s ancestral graves cannot be separated from the debates about their land, their health and their future.

## Land degradation and environmental pollution

While the state, business people and construction companies saw the N2 Toll Road project as the tool for economic transformation in the Wild Coast, the majority of local communities and environmental advocacy groups were concerned that the project would pose substantial environmental risks. Commenting on this, one of the senior BSSA directors in Msikaba said that:

‘Trees will be destroyed and deforestation will take place in the process of opening the space for the road and there are some special trees that we try by all means to conserve and they are likely to be destroyed in the process. In other words, the road will cause environmental degradation because also sand from the ground will be used in for the construction of the road.’ (Interview with Participant E6, 12 August 2021)

Furthermore, some environmental experts argued that road construction is harmful to nature in a variety of ways. During an interview in Port St. Johns, one of BSSA’s environmental experts, roughly 34 years old, explained thus:

‘The road will be harmful to nature. For example, the road will disturb fish and other sea creatures because of noise from high sound construction vehicles and construction activities such as rock blasting among other. Species like fish do not want noise at all. They run away from the shores to the deeper parts of the ocean, meaning that fishing will be impossible. Besides, the construction companies are establishing construction sites very close to the ocean and now oil and other liquids like fuel from the vehicles will pollute the water from the thus destroying natural habitats.’ (Interview with Participant E7, 06 August 2021)

During a public consultation exercise organised by Ferret Mining and Environmental Services^7^ (Pty) Ltd on 22 July 2019 that the researchers attended, it was noticed that:

‘It is considered that chemical pollution from exhaust fumes, oil spillage and accumulation of rubber compounds from tyre wear during the operational phase of the proposed toll highway would result in potential impacts of medium intensity and significance without and with mitigation. This would be of particular importance at the proposed interchanges and Ndwalane mainline toll plaza location. It is considered unlikely that the intensity and significance of the potential impact could be reduced.’ (Ferret Mining and Environmental Services Public consultation exercise, 22 July 2019)

Continuing, the Ferret Mining and Environmental Services (Pty) Ltd stressed:

‘The operation of the proposed toll highway in the Greenfields section of the Wild Coast would result in noise from vehicle traffic and at night would also involve considerable light pollution from vehicle headlights. Cumulatively, these factors could depress local faunal populations. Furthermore, it is expected that the potential direct, indirect and cumulative impacts of the construction and operation of the proposed Greenfields section between Ndwalane and the Ntafufu River would, overall, probably result in disruption of biological interactions which, in turn, may lead to a resultant loss or change of ecosystem function for example nutrient cycling or interruption of ecological processes.’ (Ferret Mining and Environmental Services Public consultation exercise, 22 July 2019)

One of the top managers of environmental affairs in Bizana explained that the road has many devastating impacts on the environment. Among the major impacts mentioned by the manager are soil erosion, increased runoff and drainage, sedimentation and silt loads. The manager explained:

‘Activities such as site clearing, increasing pavement areas will result in increased drainage and runoff, soil erosion, sedimentation and silt loads. Stern measures should be taken to minimise and restrict clearing of areas required for construction purposes only. Site offices should be situated at the right places where they do not disturb vegetation and natural habitat.’ (Interview with Participant C7, 06 August 2019)

In summary, the given environmental concerns that were raised by the participants show their objections to the N2 Toll Road project as well as SANRAL’s preferred route. The route that SANRAL chose is said to have a devastating impact on the Pondoland endemic species, natural habitat and land for agriculture among other key environmental issues. The participants’ views concur with the CCA Environmental Report (2005), which indicates that the route will have impacts on vegetation, aquatic systems, topography, agriculture, and soils and cultural, archaeological and paleontological sites. Similarly, the BSSA and the WESSA argue that the road will negatively affect ecotourism ventures in the region and will further endanger an already fragile local economy (CCA Environmental Report 2005).

However, other local commentators and environment experts in the region who support the road project argued that the environmental consequences attached to it were being exaggerated. They did not agree with some of the issues raised by the environmental advocacy groups on the potential risks of the N2 Toll Road project. They argued that some of the consequences are just rhetoric and theoretical. Furthermore, they argued that various mitigation measures are being put in place in response to the potential risks and impacts.

## Discussion

The findings of the study show that the majority of local communities, and environmental advocacy groups in the study area hold various risk perceptions on the N2 Toll Road. These risk perceptions have divided the stakeholders into different world views or solidarities as explained by the CT of risk perception. In order to demonstrate the deeply divided positions of different solidarities on policy issues, Thompson (2003) cites a 19th century narrative of Reverend Sidney Smith who observed two women arguing uncompromisingly from two opposing positions: ‘They will never agree’, said the 19th century wit, the Reverend Sidney Smith, when he saw two women shouting at each other from houses on opposite sides of an Edinburgh street, ‘they are arguing from different premises’. Different premises concern human beings and physical nature, and CT maps them in terms of the fourfold typology of social solidarity or worldviews.

For instance, local communities Wild Coast, as portrayed by fatalist world view, presume that the road construction will not benefit them, but will rather alter their way of life as it will open access to natural resources, lead to loss of land, livestock, and environmental degradation, among others. On the other hand, the environmental advocacy groups as portrayed by the egalitarian world view presume that the N2 Toll Road would cause environmental degradation and destroy rare plant species in the Wild Coast. Business people in the Wild Coast as portrayed by individualist world view on one hand, believe that the road would make their businesses thrive, they also believe that the road would bring competition in business, leading to their downfall as huge corporations are likely to appear for investment opportunities. Lastly, the government as portrayed by the hierachists’ worldview, holds the view that although there are risks associated with the road project, nature can be controllable through sound environmental policies hence minimising risks. Therefore, the hierachists, accept information from the egalitarians who are typically environmental experts, and vice versa. For instance, the government of South Africa relies on the information from the Department of Environment, Forestry and Fisheries about the environmental precautions to take on the N2 Toll Road project. This suggests that there might be cross-orientation of alliances among stakeholders in some cases. For example, because the orientations of individualists (business people) and hierarchists (the state) have similar economic interests in the exploitation of resources, they are most likely to be related to each other. By contrast, as fatalists (local community members) and egalitarians (environmental advocacy groups) share similar interests in protecting the environment, they are likely to align themselves with an individualistic or hierarchical alliance that is profit seeking from environmental disturbances (Thompson 2008).

In terms of the CT, conflict among the stakeholders involved in the N2 Toll Road project stems from their risk perceptions linked to loss of land, loss of habitat and rare plant species, destruction of sacred aquatic environments, cultural spaces and grave removal, noise and environmental pollution as well as power imbalances. Various studies, including Brenot et al. (1998) and Yuan et al. (2019), have corroborated the CT’s assertion that there is a strong link between cultural world views and risk perception.

However, to harmonise different groups as represented by the worldviews embedded in the CT, Verweij, Michael Thompson and colleagues (eds. 2006) suggest the clumsy solution as a way forward. The clumsy solution originated from the CT, and it put forward that the divergent discourses, narratives and storylines of each of the stakeholders can only be accommodated by a ‘clumsy solution’, or ‘pluralistic model’, which combines the contradictory interests and voices of the stakeholders (eds. Verweij & Thompson 2006). Its main objective is to provide a new solution framework to the wicked problems of spatial planning. In other words, clumsy solutions mean that we should not look for ideal solutions to ambiguous, dynamic and normative problems, but rather that we should look for feasible solutions that respond to various rationalities. This process is described by Verweij and Thompson (2006) as ‘negotiated agreement’ among stakeholders and can be achieved through ensuring legitimacy of the plan and promoting democracy through grassroots participation particularly in the case of the N2 Toll road project under study.

## Conclusion

Despite the post-apartheid government’s claim that the N2 Toll Road project is essential for the ‘public good’, it overlaps with areas of high and important biodiversity. As a result, attempts to implement such a land-based poverty-reduction project lead to clashes among individuals and groups ostensibly concerned with the welfare of local people and environmental protection. The clashes as highlighted in the background have existed since project planning in the late 1990s and have delayed project completion because of risk perceptions among stakeholders. In this regard, the CT provides a clear understanding of the cultural preferences of different stakeholders as well as their reactions to risks and vulnerabilities. Most importantly, the CT suggests the ‘clumsy solution’ as the way forward to harmonize the different conflicting groups in order to reach the goals of economic growth and environmental sustainability in the Wild Coast. In the case of the N2 Toll Road project, contradictions in the stakeholders’ risk perceptions are unabated because of their differences in cultural adherences, which frequently lead to socio-environmental conflicts. These conflicts are exacerbated by a lack of community participation and the use of state power to capture rights or neglect the environmental interests of the people at the grassroots level.

## Recommendations

Based on the findings of this study, we recommend that the ecological effects of multi-scale infrastructure projects such as roads, railways and dams should be examined. Therefore, modern technology such as remote sensing, data compilation, analysis and modelling should be used for road alignments to determine the potential socio-environmental impacts.

Similarly, as the study has revealed that many residents of the Wild Coast communities practice a variety of cultural rituals and hold sets of beliefs in their daily lives, it is critical to recognise the significance of their cultural spaces and aquatic environments for community cultural health in infrastructure development and environmental policy discourse.

## Footnotes

1 It is a development strategy stated in the Eastern Cape Growth Province, with the primary goal of generating long-term economic growth and development in the Wild Coast region. It also focuses on providing local residents with stable and long-term employment (Goliath, Mxunyelwa & Timla 2018).

2 Apartheid refers to ‘a policy of segregating people by race in terms of where they lived, where they went to school, where they worked, and where they died. It literally means “apartness or separateness” in Afrikaans and Dutch. This policy was implemented in South Africa by the National Party government in 1948, and it remained in effect until its demise in 1994’ (Clark & Worger 2022:6).

3 A major trade corridor connecting South Africa’s Gauteng, Mpumalanga, and Limpopo provinces with Maputo, the capital and port of Mozambique.

4 ‘A legal and institutional framework, facility and associated procedures that enable goods, people and vehicles to stop in a single facility in which they undergo necessary controls following applicable regional and national laws to exit one State and enter the adjoining State’ (Muqayi & Manyeruke 2015:11).

5 Pondoland is a region on the South African coast of the Indian Ocean. It is situated on the coastal belt of the Eastern Cape province. Its jurisdiction is the former Pondoland and the traditional region of the Pondo people, one of the sub-groups within the Xhosa ethnic nation.

6 A practitioner of ngoma, a philosophy based on a belief in ancestral spirits (Zulu, Sesotho, Xhosa: Izinyama, siSwati and amadlozi among others). The Sangomas are regarded as potential sources of social and political healing (Wreford 2005).

7 The company is working in Environmental Services business activities.
